# A Small Molecule (Pluripotin) as a Tool for Studying Cancer Stem Cell Biology: Proof of Concept

**DOI:** 10.1371/journal.pone.0057099

**Published:** 2013-02-21

**Authors:** Susan D. Mertins, Dominic A. Scudiero, Melinda G. Hollingshead, Raymond D. Divelbiss, Michael C. Alley, Anne Monks, David G. Covell, Karen M. Hite, David S. Salomon, John E. Niederhuber

**Affiliations:** 1 Screening Technologies Branch, Developmental Therapeutics Program, Division of Cancer Treatment and Diagnosis, National Cancer Institute at Frederick, Frederick, Maryland, United States of America; 2 SAIC-Frederick, National Cancer Institute at Frederick, Frederick, Maryland, United States of America; 3 Biological Testing Branch, Developmental Therapeutics Program, Division of Cancer Treatment and Diagnosis, National Cancer Institute at Frederick, Frederick, Maryland, United States of America; 4 Mammary Biology and Tumorigenesis Laboratory, Center for Cancer Research, National Cancer Institute, Bethesda, Maryland, United States of America; 5 Cell and Cancer Biology Branch, Center for Cancer Research, National Cancer Institute, Bethesda, Maryland, United States of America; Sun Yat-sen University Cancer Center, China

## Abstract

**Background:**

Cancer stem cells (CSC) are thought to be responsible for tumor maintenance and heterogeneity. Bona fide CSC purified from tumor biopsies are limited in supply and this hampers study of CSC biology. Furthermore, purified stem-like CSC subpopulations from existing tumor lines are unstable in culture. Finding a means to overcome these technical challenges would be a useful goal. In a first effort towards this, we examined whether a chemical probe that promotes survival of murine embryonic stem cells without added exogenous factors can alter functional characteristics in extant tumor lines in a fashion consistent with a CSC phenotype.

**Methodology/Principal Findings:**

The seven tumor lines of the NCI60 colon subpanel were exposed to SC-1 (pluripotin), a dual kinase and GTPase inhibitor that promotes self-renewal, and then examined for tumorigenicity under limiting dilution conditions and clonogenic activity in soft agar. A statistically significant increase in tumor formation following SC-1 treatment was observed (p<0.04). Cloning efficiencies and expression of putative CSC surface antigens (CD133 and CD44) were also increased. SC-1 treatment led to sphere formation in some colon tumor lines. Finally, SC-1 inhibited in vitro kinase activity of RSK2, and another RSK2 inhibitor increased colony formation implicating a role for this kinase in eliciting a CSC phenotype.

**Conclusions/Significance:**

These findings validate a proof of concept study exposure of extant tumor lines to a small molecule may provide a tractable in vitro model for understanding CSC biology.

## Introduction

Cancer stem cells (CSC) are an area of considerable interest to cancer biologists and thought to be responsible for the long-term maintenance and expansion of both solid and hematologic tumors [1], [2]. Under the cancer stem cell hypothesis, CSC may explain the observed tumor heterogeneity that is reminiscent of normal organ development and the resistance of cancer to standard therapies [3]. Signaling pathways such as those modulated by Wnt, Hedgehog, and Notch are implicated in the biochemical characterization of CSC but are not consistently found in all tumor types. Furthermore, the roles of the tumor microenvironment in regulating CSC continue to be an area of intense study [4].

Several functional assays are understood to represent the self-renewal, proliferation, and differentiation capacities expected of CSC. First, limiting dilution tumorigenicity assays represent a standard method for identifying CSC. This in vivo model utilizes immunodeficient mice inoculated with tumor cell subpopulations that have been selected for expression of normal tissue stem cell surface antigens [5]–[7]. Using the same model, secondary xenografts can be established and reexamined for transmission of the original tumor heterogeneity, thus further confirming the presence of CSC. A second assay uses sphere formation to select for CSC expressing stem cell markers and tumorigenicity [8]. Thirdly, clonogenic activity in soft agar has been used to define CSC [9]. Other assays available for characterizing CSC phenotypes focus on measures of drug resistance, quiescence, and resistance to apoptosis [10].

In some tumor types, CSC are expected to be a rare subpopulation and the means to maintain them in culture does not exist at present. A complicating factor reported by Gupta et al. demonstrates that after enriching for and culturing a CSC-like subpopulation in a breast tumor line, a phenotypic equilibrium containing mixed subpopulations returns [11]. Expressed genotypes, however, can be used to predict the operational signal transduction pathways that establish a CSC phenotype. Modulation of those relevant pathways by chemical probes offers a means to better understand CSC biology. Based on this rationale, we examined whether a small molecule, SC-1 (pluripotin), could modify and/or induce characteristics consistent with the CSC phenotype in the seven colon tumor lines of the NCI60 Tumor Line Panel.

SC-1 was discovered in a cell-based high throughput screen that assessed whether a small molecule could maintain self-renewal of normal murine embryonic stem cells in the absence of exogenously added factors such as leukemia inhibitory factor or feeder cells [12]. The chemical structure is based on a 3,4-dihydropyrimido(4,5-d)pyrimidine scaffold and was optimized through structure/activity studies. Affinity chromatography established that molecular targets for SC-1 include RasGAP and ERK1/2, and their inhibition is thought to promote self-renewal and inhibit differentiation.

In this report, we offer a proof of concept study to determine whether SC-1 treated bulk cell populations of the NCI60 colon tumor lines possess characteristics consistent with a CSC phenotype. Our findings provide the basis for pursuing further studies that could confirm the presence of CSC in a tractable in vitro model, promote understanding of the biochemical pathways that underlie CSC phenotypes, and determine if SC-1 is a useful tool for maintaining tumor biopsy-derived CSC in culture.

## Results

### SC-1 Increased Tumor Formation

We pretreated bulk populations of the seven colon tumor lines of the NCI60 panel with 0.1 µM SC-1 (for chemical structure see Figure S1), a small molecule previously shown to promote self-renewal in murine embryonic stem cells [12]. After five days of treatment, limiting dilution tumorigenicity studies were conducted utilizing subcutaneous injections without any protein support (Table 1). Evaluating all tumor lines from a single subpanel of the NCI60 will help determine whether any effect due to SC-1 is universal or tumor line-dependent. Five of seven colon tumor lines exhibited a higher tumor take rate for SC-1 treated lines compared to control at the 10,000 cell inoculum, with COLO 205 tumor line demonstrating the largest difference in take rate (control: 1 of 5 vs. SC-1 treated: 4 of 5) followed by HCT-116 and HT29 tumor lines. It is also important to note that with as few as 100 cells, tumors formed in 2 of 5 mice injected with SC-1 treated HT29 cells compared to 0 of 5 for control treated cells. There was a statistically significant increase in tumor formation at the 1000 cell inoculum for HT29 treated cells as well (Table 1). In contrast, no tumors developed with the HCT-15 tumor line at any dilution (Table 1), although tumors formed in 16 days at a routine inoculation size (1.5×10^6^ cells per inoculum).

**Table 1:** Effect of SC-1 on the Tumor Take Rate of the Seven Colon Tumor Lines of the NCI60 Panel*.

	Number of Cells Inoculated					
Cell Line	100		1000		10000	
	Control	SC-1	Control	SC-1	Control	SC-1
**COLO 205**	0/5	0/5	1/4	1/5	1/5	4/5
**HCC-2998**	0/5	0/5	0/5	0/5	2/5	2/5
**HCT-15**	0/5	0/5	0/5	0/5	0/5	0/5
**HCT-116**	1/5	0/5	3/5	2/5	3/5	5/5
**HT29**	0/5	2/5	0/5	3/5@	3/5	5/5
**KM12**	0/5	0/5	0/5	0/5	0/5	1/5
**SW-620**	0/5	0/5	1/5	0/5	1/5	2/5
**TOTAL**	**1/35**	**2/35**	**5/34**	**6/35**	**10/35** &	**19/35**

* Female NOD.SCID mice were inoculated s.c. with bulk population colon cell lines treated for 5 days as indicated with control media or media containing 0.1 µM SC-1.

@Significant difference between control and SC-1 treated groups in the HT29 colon tumor line, 0.0375<p<0.05.

& Significant difference between control (10/35) and SC-1 (19/35) treated group for 7 colon tumor lines, p = 0.04.

Because combining the tumor take rate (at the 10,000 cell inoculum) might resemble a clinical study with patient tumor heterogeneity, a statistical analysis was performed to evaluate the effect of SC-1 on the tumor take rate of the colon tumor line subpanel. A significant difference was found (n = 7, Cochran Mantel-Haenzel common odds ratio, control tumor take rate: 10 of 35 mice; SC-1 treated take rate 19 of 35 mice, p = 0.04). In addition, these same results are replotted in graphic form in Figure S2. The frequency of tumor initiating cells was also calculated and presented for all tumor lines and the most SC-1 sensitive cell lines in Figure S3.

The data showing increased tumor take rate were complemented by the accelerated appearance of measureable tumors for the SC-1 treated colon tumor lines when calculated from plotting average tumor weight (up to 2,000 mg) vs. time for each treatment group (range of r for fitted lines: 0.73–0.90). Extrapolation was used to determine the day at which a 1000 mg tumor would be expected (Table 2). The results and subsequent calculations found that 1000 mg tumors, on average, formed after pretreatment with SC-1 in less than half the time of the control treated group (n = 5, paired Student’s t test, mean±s.e.m., SC-1 treated: day 83±19 vs. control treated: day 211±79). Although this difference was not statistically significant (p = 0.16), it is notable, that for these 5 tumor lines, the trend was the same: earlier time to 1000 mg tumor appearance for SC-1 treated cells vs. controls.

**Table 2:** Calculated Time to 1000 mg Tumor Volume in 5 Colon Lines Forming Tumors*.

	Day#	
Cell Line	Control	SC-1
COLO 205	513	101
HCC-2998	184	139
HCT-116	48	45
HT29	185	32
SW-620	126	98

* NOD.SCID mice injected s.c. with treated colon tumor lines at 10,000 cells/inoculum. Linear curves were fitted to population averages plotted for time (up to 120 days) vs. calculated mean tumor size (range of r: 0.73–0.90). Because mice injected with KM12 treated cells formed only one tumor in SC-1 conditions and no tumors under control conditions, this result was excluded from the analysis. Colon tumor lines were treated with 0.1 µM SC-1 or control media five days prior to inoculation.

# Mean day±S.E. was 211±79 for control vs. 83±19 for SC-1 treated (n = 5, p = 0.16, Student’s paired t test).

It is not likely that the observed SC-1 induced accelerated *in vivo* tumor growth rate could explain the increased tumor take rate of SC-1 treated tumor lines because a statistically significant decrease in cell number after *in vitro* SC-1 treatment was observed. (n = 6, paired Student’s t test, p = 0.02, Table S1). Additionally, the mean GI_50_ for the colon cell lines, when evaluated in the NCI Anticancer Screen (2 day exposure), was 0.091±0.07 µM (mean±s.e.m., n = 7, GI_50_ (concentration at which the compound inhibits 50% of control cell growth)), suggesting a uniform *in vitro* growth inhibition amongst the tumor lines. Finally, in a representative SC-1 sensitive tumor line (HCT-116), there was no difference in the distribution of the SC-1 treated cell population across the cell cycle compared to control treated cells (Figure S4).

**Figure 1:**
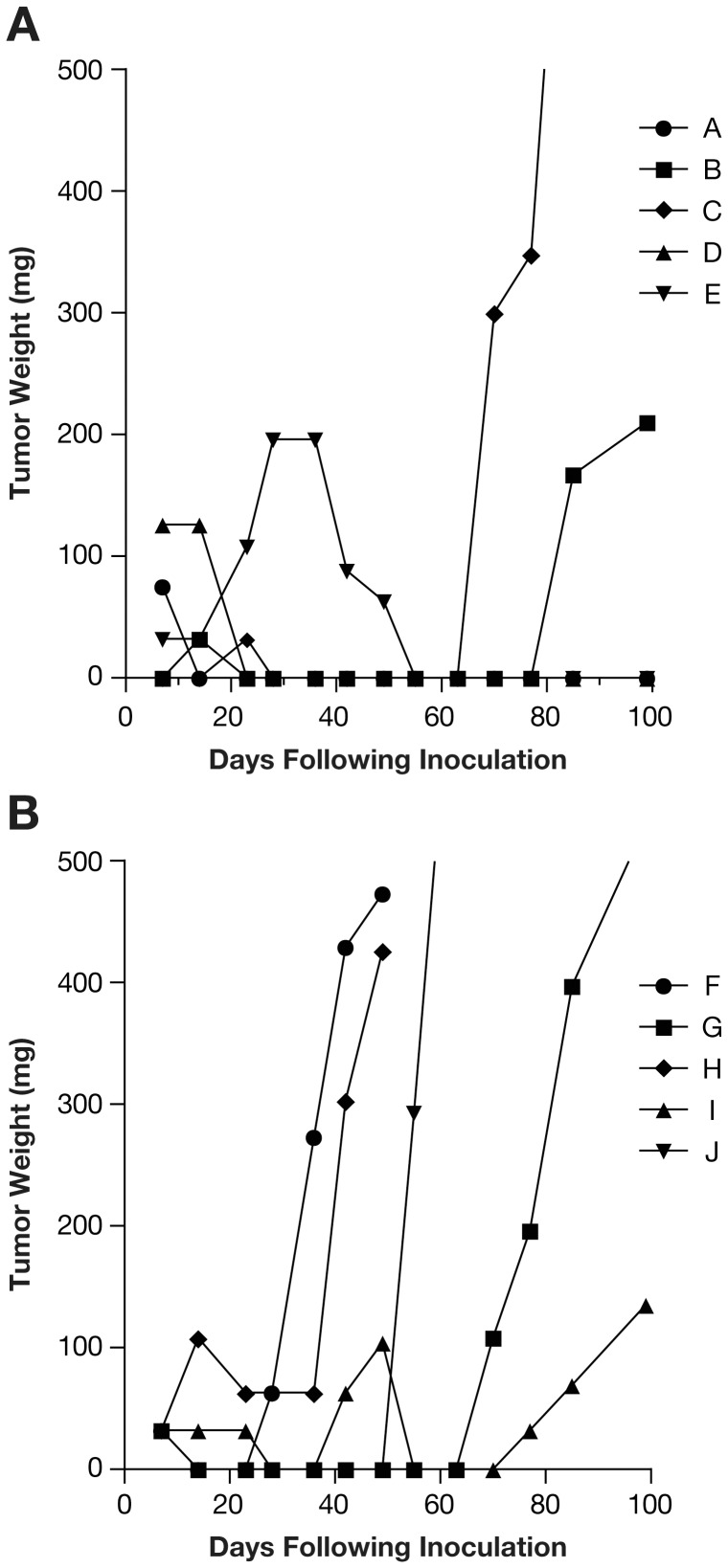
Effect of SC-1 on Tumor Formation in HCT-15 Colon Tumor Line with a 10 Cell Inoculum and Matrigel Co-injection. Limiting dilution tumorigenicity assay was performed with simultaneous injection of Matrigel. A. Control treated HCT-15 tumor line. B. SC-1 treated HCT-15 tumor line. Tumor weight for each mouse is depicted. Tumor formation was increased in SC-1 treated cells when 10 cells/mouse were injected (SC-1 treated: 5 of 5 tumors formed, Control treated: 2 of 5 tumors formed). At higher cell inocula, all mice formed tumors independent of treatment. Each line in both graphs represents growth of one tumor per mouse (A. A–E, B. F–J).

To explore the lack of a tumorigenic effect for SC-1 treated HCT-15 cells, the limiting dilution tumorigenicity assay was repeated with the addition of Matrigel to the injected cells (10, 100, 1000, and 10000 cells per injection). A protein support such as Matrigel may provide anchoring, a substrate for angiogenesis, and growth factors. In this case, as seen previously [7], as few as 10 cells were needed to form tumors (take rate 2 of 5 mice) when HCT-15 control cells were co-injected with Matrigel. For SC-1 treated cells co-injected with Matrigel, 5 of 5 mice had tumor formation over the 99 day observation period at the 10 cell inoculum (Figure 1). At the remaining cell inocula (100, 1000, and 10000 cells per injection with Matrigel), tumors formed in all mice, regardless of experimental treatment.

### Cloning Efficiency was Increased Following SC-1 Treatment of Colon Tumor Lines

**Figure 2:**
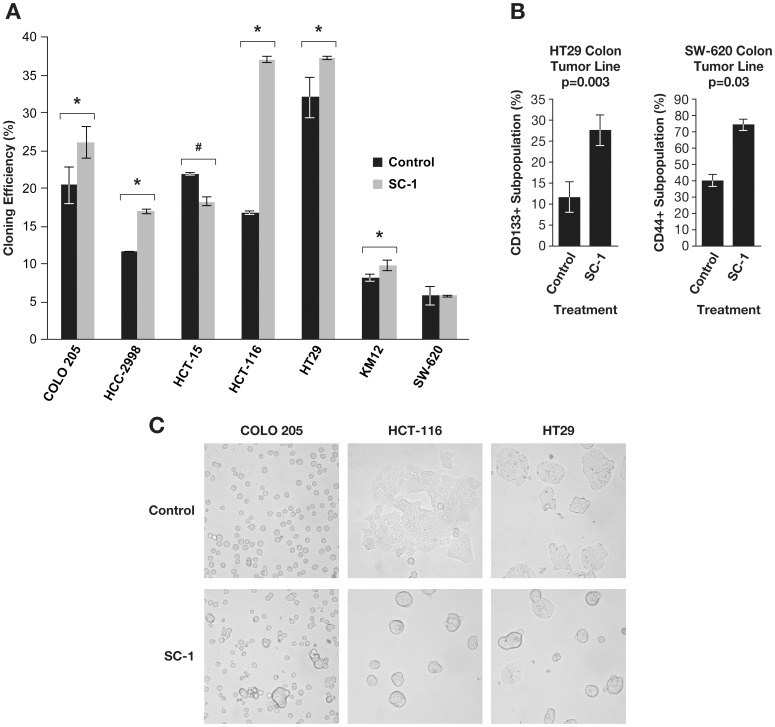
Tumor Lines Treated with SC-1 Possess Characteristics Consistent with the CSC Phenotype. The colon tumor lines were treated for five days with 0.1 µM SC-1, harvested and then analyzed for their ability to form colonies in soft agar, to alter expression of putative CSC surface markers, and to form spheres. A. The cloning efficiency of the colon tumor lines was determined following treatment with SC-1. In 5/7 tumor lines, SC-1 treated cells had statistically significant increases in cumulative colony forming unit (CFU) mass compared to control treated cells (***p<0.001, **p<0.1 *p<0.05, n = 3). In one instance (#p<0.05), SC-1 treatment reduced cloning efficiency. B. The CD133 positive subpopulation was increased 2.5-fold in SC-1 treated HT29 colon tumor line (p = 0.03, n = 3). The CD44+ CD24- subpopulation was increased after SC-1 treatment in the HCT-116 colon tumor line (*p<0.05, n = 3). The CD44+ subpopulation was increased significantly following SC-1 treatments (*p<0.05, n = 3) in SW-620 colon tumor line. C. Three tumor lines (COLO 205, HCT-116, HT-29) formed nonadherent spheres in the presence of 0.1 µM SC-1 cultured in standard media containing 5% FBS. These observations were also apparent at day 5 in culture. All images were prepared at 400× magnification.

Because enhanced clonogenic capacity in soft agar has been linked to CSC derived from patient CNS and prostate tumors [9], [13], it was of interest to evaluate if SC-1 treated colon tumor lines were similarly affected. The 3 tumor lines with the largest SC-1 induced tumor formation (COLO 205, HCT-116, and HT29) had significant increases in cloning efficiencies (Figure 2A, n = 3, paired Student’s t test, p = 0.001 (COLO 205, HCT-116) and p = 0.01 (HT29)) as did HCC-2998 and KM12 tumor lines. It is notable that all tumor lines had increased cloning efficiency following SC-1 treatment if the assay was conducted using Matrigel instead of soft agar (data not shown). Thus, SC-1 is found to enhance colony formation *in vitro*.

### Expression of Putative CSC Markers were Increased in Colon Tumor Lines following SC-1 Treatment

Monoclonal antibodies specifying surface antigens on freshly isolated tumor samples have been utilized as tools to isolate CSC. For example, O’Brien et al. [6] and Ricci-Vitiani et al. [14] purified CSC from colorectal tumors via the glycosylated CD133 epitope. Other CSC markers include CD44 (either in the presence or absence of CD24), CD326, and CD166 [5], [9], [15]–[18]. Thus, the expression of these markers was examined following five days of SC-1 treatment.

A statistically significant increase in the number of cells expressing the CD133 glycosylated epitope was found for the HT29 tumor line following SC-1 treatment (Figure 2B, n = 3, paired two tailed Student’s t test, p = 0.003, mean±s.e.m, control treated: 11.6±3.7% positive, SC-1 treated: 27.6±3.6% positive). In the SW-620 tumor line, the CD44 subpopulation was increased following treatment with SC-1 (Figure 2B, n = 3, paired Student’s t test, p = 0.03, mean±s.e.m., control treated: 39.6±8.5% positive, SC-1 treated: 74.1±13.4% positive). No increased expression of the putative CSC markers was evident for the COLO 205 tumor line (a SC-1 sensitive tumor line). No changes in CD326 and CD166 surface expression were observed for any of the tumor lines. Thus, change in expression of certain CSC surface markers occurred following SC-1 treatment in colon tumor lines varied with the tumor line.

### Spheres Formed Following SC-1 Treatment

Sphere formation is a CSC characteristic, whether derived from patient tumors [18] or extant tumor lines of the brain, breast, or skin grown under serum free conditions [8]. SC-1 treated colon tumor lines were examined for sphere formation utilizing cell densities previously published [19], [20] and in the presence of serum. Because serum is thought to contain differentiating agents [21], this assay might be considered more stringent than others [22]. In the HCT-116 and HT29 tumor lines, uniform nonadherent spheres with well-defined borders formed in 24 hrs (Figure 2C) despite the presence of serum. Additionally, sphere formation occurred in a fraction of the cultured COLO 205 tumor line (Figure 2C). These same alterations in morphology were also present at day 5 following treatment (data not shown). In the remaining tumor lines, sphere formation was found at concentrations higher than those tested here (data not shown). The sphere formation assay was repeated utilizing serum free conditions and similar results were obtained (Figure S5).

### SC-1 Treatment Decreased Phospho-ERK1/2 and Increased OCT4 Protein Expression Levels

**Figure 3:**
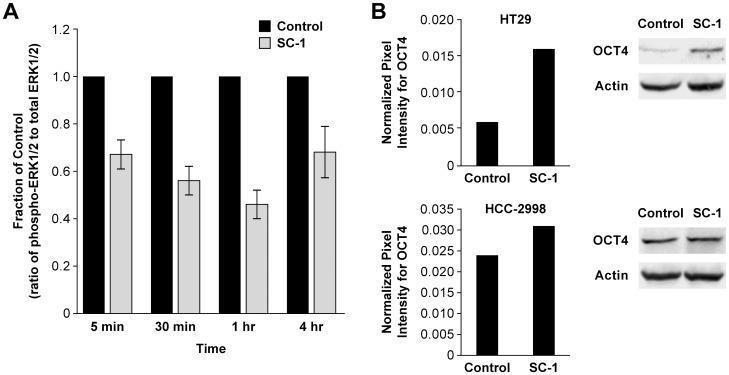
Effect of SC-1 on Protein Expression Levels of phospho-ERK1/2, and OCT4 in Colon Tumor Lines. Colon tumor lines were treated with SC-1 (0.1 µM), harvested, lysed, and probed for the proteins of interest following electrophoresis in SDS-PAGE gels at the indicated time points. A. In the SC-1 treated HT29 tumor line, phospho-ERK1/2/total ERK1/2 protein levels were decreased to 67±0.06% of control value at 5 min, 56±0.06% of control value at 30 min, and 46±0.06% of control value at 1 hr (n = 3, p<0.05). B. Increased OCT4 protein expression due to SC-1 was dependent on the tumor line. Representative experiments are shown for Figure S4A–B (n = 2).

A previous report [12] demonstrated that SC-1 inhibited phospho-ERK1/2 protein levels after 30 minutes of exposure in murine embryonic stem cells resulting in maintenance of self-renewal *in vitro* without lymphocyte inhibitory factor or feeder cells. To determine if SC-1 reached the same target in the treated colon tumor lines, changes in abundance of phospho-ERK 1/2 (p-ERK) and total ERK1/2 protein were evaluated by immunoblotting at time points similar to those previously studied (Figure 3A). In the HT29 treated tumor line, phospho-ERK 1/2 protein levels (relative to total ERK1/2 protein levels) were decreased in 5 min (67±0.06% of control value), reached a trough at 1 hr (46±0.06% of control), and remained decreased during the remainder of the time course (4 hrs, 68±0.10% of control value). Thus, it is likely SC-1 is reaching its cognate molecular target in the HT29 tumor line.

Increased levels in OCT4 protein expression in SC-1 treated colon tumor lines would be consistent with the identification of SC-1 in a cell-based screen that detected maintenance of an OCT4-mediated GFP signal in mouse embryonic stem cells growing without exogenous factors or feeder cells [12]. OCT4 is a transcription factor important for embryonic development and regulates pluripotency [23]. Therefore, changes in OCT4 protein levels following exposure to SC-1 were evaluated. In 2 of the 7 colon tumor lines (HCC-2998 and HT29), OCT4 protein expression was increased with SC-1 treatment (Figure 3B). For the remaining tumor lines, OCT4 protein expression was unchanged or decreased. The variable effect of SC-1 on OCT4 protein expression did not correlate with tumor formation. Therefore, it is unlikely OCT4 plays an important role in the observed SC-1 effects.

### SC-1 Inhibited RSK2 *In Vitro* and a RSK2 Inhibitor Increased Colony Formation

**Figure 4:**
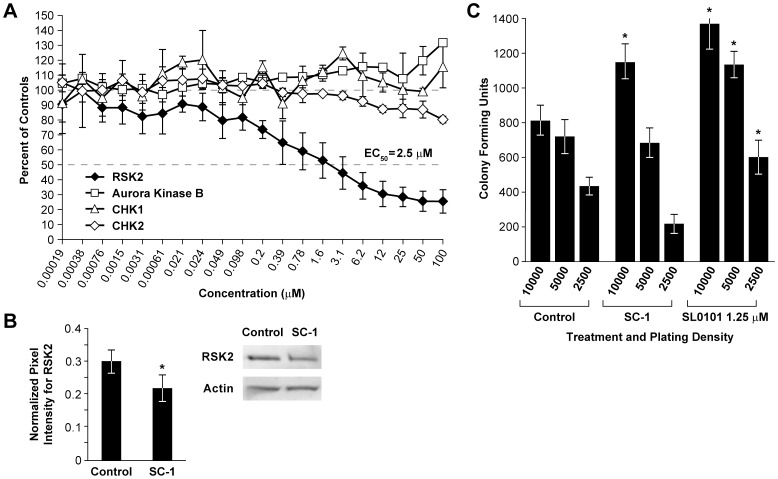
SC-1 Inhibited RSK2 Kinase Activity *in vitro* and an RSK2 Inhibitor Increased Colony Formation. COLO 205 tumor cell line was treated with SL0101 (1.25 µM) and SC-1 (0.1 µM) for 24 hrs prior to soft agar cloning, lysate preparation, and immunoblotting. A. SC-1 inhibited RSK2 N-terminal domain *in vitro* kinase activity with an EC_50_ of 2.5±1.8 µM (mean±SD, n = 5) but not in other like assays for Aurora kinase B, CHK1, or CHK2 kinases. B. Decreased RSK2 protein levels (33%) were found at 5 days following SC-1 treatment in the COLO 205 tumor line (n = 2). C. Treatment (24 hrs) of COLO 205 tumor line with SL0101, a kaempferol glycoside RSK2 inhibitor, increased colony formation (≥60 µm) in soft agar (n = 2, representative experiment shown).

A bioinformatic pathway analysis [24], [25] of genes that correlated with the NCI60 GI_50_ fingerprint for SC-1 suggested a role for RSK2 and its regulatory activity in the mTOR pathway. RSK2 (90 kD ribosomal S6 kinase), a kinase with both a N- and C-terminal functional domains, phosphorylates multiple targets and is phosphorylated by ERK1/2, another SC-1 target. As a downstream effector, RSK2 signals many cellular behaviors including cell survival, growth, proliferation, and migration [26]. It is also notable that a congener of SC-1 has been co-crystallized with Bcr-ABL1 kinase [27], suggesting that SC-1 may have other molecular targets. We examined whether SC-1 could inhibit the *in vitro* kinase activity of RSK2 and found an EC_50_ of 2.5±1.8 µM (Figure 4A, EC_50_, concentration at which the compound inhibits 50% of control activity, n = 5). Potential inhibitory activity of SC-1 on a random selection of other protein kinases (Aurora kinase B, CHK1, and CHK2) was also evaluated in the same assay and no effect was found (Figure 4A). To determine if activity of related (to RSK2) protein kinases in the AGC kinase family was inhibited by SC-1, further studies were conducted (Table 3). No inhibitory effect on kinase activity was found for Akt1, PKA, and PKC at or below the maximum concentration tested (10 µM); however, SC-1 was inhibitory for p70S6K (1.4 µM IC_50_). Because SC-1 inhibited these selected kinases in the micromolar range and the kinase assay is distinct from the one reporting data in Figure 4A, the positive EC_50_ value for Abl1 kinase was reported in Table 3 as well. These findings were consistent with predictions reported by Okram et al. [27].

**Table 3:** Effect of SC-1 on *In Vitro* Kinase Inhibition in Selected Kinases*.

Protein Kinase	IC_50_ (µM)
Abl1	0.005
Akt1	–
PIM1	–
PKA	–
PKC	–
p70S6K	1.4
PLK2	2.2
RSK1	0.5
RSK2	2.5
RSK3	3.3
RSK4	10.0

* SC-1 was evaluated for *in vitro* kinase inhibition utilizing a miniaturized ^33^P-based screening assay [41]. The maximum concentration tested was 10 µM. IC_50_ (inhibitory concentration of 50% of the control (DMSO) treatment) was calculated from a 3-fold serial dilution (n = 2). Dashed lines indicate no effect at or below the maximum concentration tested.

Since SC-1 inhibited RSK2 kinase activity, we first determined if RSK2 protein levels were altered. After 5 days of exposure to 0.1 µM SC-1, RSK2 protein levels were significantly decreased 33% in COLO 205 tumor line (p = 0.007, n = 3, Figure 4B).

If RSK2 plays a role in cell signaling essential for the CSC phenotype, it would be expected that enhanced colony formation would occur following treatment with a known RSK2 inhibitor. To that end, COLO 205 tumor line was treated with SL0101, a kaempferol glycoside with no activity against MEK, Raf, or PKC [28], for 24 hrs prior to cloning in soft agar and evaluated for colony formation 7 days later. Statistically significant increases in colony formation were found at 1.25 µM treatment and at all plating densities (Figure 4C, n = 3, paired two tailed Student’s t test, p = 0.05). For SC-1 treated cells at this shortened exposure (24 hrs (Figure 4C) vs. 5 days (Figure 2A)), increased colony formation was found only at the highest plating density.

## Discussion

Bulk populations of colon tumor lines from the NCI60 Panel were exposed to a small molecule that confers self-renewal properties and examined for characteristics consistent with the CSC phenotype. Pretreatment with SC-1 increased tumorigenicity at low cell inocula in HT29 and HCT-15 tumor lines. Cloning in soft agar was increased in most tumor lines. The proportion of cells expressing CD133 and CD44, putative CSC surface antigens, was increased. Sphere formation in response to SC-1 treatment was also observed under serum containing and serum free conditions. To confirm that SC-1 was altering its cognate molecular target, protein expression of phospho-ERK1/2 was measured and found to decrease after 5 min and up to 4 hrs after exposure. Expression of OCT4, a transcription factor that maintains pluripotency in normal embryonic stem cells, was increased in some (2/7) of the tumor lines after SC-1 treatment. SC-1 inhibited RSK2 *in vitro* kinase activity. A result consistent with this finding, another RSK2 inhibitor enhancing clonogenicity of a colon tumor line, implicated this protein as a candidate molecular target mediating the CSC phenotype.

The findings of this proof of concept study that asks whether a small molecule can modify and/or induce characteristics consistent with the CSC phenotype in colon tumor lines support the feasibility of using a small molecule to establish an *in vitro* model of CSC biology. HT29 tumor line was shown to be the most SC-1 sensitive since tumors could be found with as few 100 SC-1 treated cells, clonogenicity was increased, spheres formed under conditions that usually limit such morphology and under conditions that were permissive, and expression of CD133 and OCT4 was increased. Thus, a number of avenues for researching CSC biology are suggested. First, SC-1 treated colon tumor line subpopulations expressing putative CSC antigens can be purified, then reexamined for characteristics consistent with the CSC phenotype to improve the *in vitro* model. It would also be of interest to determine if SC-1 can maintain a putative HT29 CSC subpopulation in long-term culture, overcoming the phenotypic equilibrium described for the unstable stem-like subpopulation identified in the SUM159 breast tumor line [11]. Alternatively, the stem-like cells found in SUM159 breast tumor line may be exposed to SC-1 to determine if the subpopulation returns to its mixed equilibrium of stem-like and non-stem-like cells or is maintained as stem-like. And finally, as originally proposed, tumor biopsy derived CSC could be treated with SC-1 to determine if its phenotype is maintained.

Clear identification of the subpopulation or subpopulations influenced by SC-1 could not be determined here because bulk populations of tumor lines were treated. To increase the likelihood of demonstrating an SC-1 effect, heterogeneous tumor lines were utilized. Thus, it is possible that either SC-1 maintained CSC characteristics preexisting in the colon tumor lines or biochemically established the functional parameters measured *de novo*. If the latter is true, the results support the notion that the CSC phenotype is plastic [29].

It is important to note that an SC-1 effect was not consistent across the seven colon tumor lines (Table 4). For example, overall tumorigenicity of SC-1 treated tumor cells was significantly increased at the 10000 cell inoculum but the degree to which this occurred depended on the tumor line and conditions of the tumorigenicity assay. HT29 treated tumor line was considered the most SC-1 sensitive cell line because only 100 cells were required to form tumors while, under the same limiting conditions (absence of a protein support), HCT-15 tumor cells were unable to form tumors, even in control conditions and the 10000 cell inoculum. The addition of Matrigel to both control and treated HCT-15 tumor line improved tumorigenicity and demonstrated an SC-1 effect at the 10 cell inoculum. Whether Matrigel was simply a trapping mechanism, a supply of growth factors, or a suitable matrix for angiogenesis is not clear at this time and awaits further study. Furthermore, the critical requirement for Matrigel for the HCT-15 tumor line to grow *in vivo* suggests that receptors such as integrins that bind extracellular matrix components and signal cell survival may be essential for studying CSC biology. It is notable that integrin receptors are putative CSC surface markers [30], [31]; SC-1 may be modulating these receptors.

**Table 4:** Summary of SC-1 Effects in the NCI60 Colon Tumor Lines.

	Tumorigenicity*	Clonogenicity&	Sphere Formation@	Change in Surface Marker Expression^
**Cell Line**				
COLO 205	Y	Y	Y	N
HCT-116	Y	Y	Y	Y (CD44+ CD24−)
HT29	Y	Y	Y	Y (CD133+)
SW-620	Y	N	Y	Y (CD44+)
HCC-2998	Y	Y	N	N
HCT-15	Y%	N	N	N
KM12	N	Y	N	N

* Y indicates increased tumor formation or increased time to appearance or both.

& Y indicates increased cloning efficiency in soft agar.

@Y indicates the presence of spheres following SC-1 treatment.

^ Y indicates increased putative surface marker expression using the panel of 6 antibodies described in the Material and Methods.

% Y indicates increased tumor formation when Matrigel was co-injected.

Importantly, though, a consistent definition of CSC surface markers does not exist [32], [33]. Putative CSC surface markers were evaluated following SC-1 exposure in the colon tumor lines and any altered expression was not universal, consistent with previous studies. However, CD133, a notable and common colon CSC marker in biopsy samples, was increased approximately 2-fold in treated HT29 tumor line. Furthermore, HT29 tumor line was the most SC-1 sensitive line with respect to tumorigenicity. Therefore, SC-1 exposed HT29 tumor line may be a suitable model for CSC that express CD133. But in summary, it remains possible that mechanistically, the SC-1 effect on CSC characteristics in general, and surface marker effect, specifically, may depend on the underlying genetic and epigenetic heterogeneity and not all tumor lines exposed to SC-1 will become useful *in vitro* models. Taken together, the data in this report further support the notion that stem-like characteristics are not only heterogeneous between tumor types, but also between tumors that arise from the same organ indicating that there is considerable plasticity in the generation of CSC.

SC-1 exposure induced nonadherent sphere formation and in nearly all cells in the HT29 and HCT-116 tumor lines at the 0.1 µM dose tested. This assay was conducted differently from previous reports [19], [20] by utilizing fetal bovine serum and tissue culture vessels that facilitate cell adherence and may be considered more stringent since serum can induce differentiation [21]. Additional studies, in which serum and adherent surfaces were removed, confirmed this finding. Taken together, these findings suggest that SC-1 may be promoting one of the necessary processes for metastasis, detachment from the primary tumor. Whether theses steps are consistent with the definition of a cancer stem cell is not clear presently, but suggests a role for SC-1 in the EMT transition [34].

It is likely that SC-1 is reaching at least one of its reported molecular targets (ERK1/2) [12] since as early as 5 min following exposure, the ratio of phospho-ERK1/2 to total ERK1/2 protein levels was decreased in the HT29 tumor line. Furthermore, the lipophilic nature of SC-1 (as measured by log P) suggested that it is able to cross the cell membrane. While MAPK signaling is expected to promote growth and its inhibition would be antitumorigenic [35], the dynamic nature of ERK1/2 inhibition and other known and unknown targets of SC-1 may promote the functional attributes we studied.

The mechanism of action of SC-1 is unknown at this time and we initiated studies utilizing the publically available data, gene expression profiles in NCI60 Anticancer Drug Screen [24], [25] to examine this. In particular, a bioinformatics pathway analysis of genes that correlated with the SC-1 NCI60 GI_50_ mean graph suggested a role for the mTOR pathway in modulating the observed growth inhibitory effects (*in vitro*) with RSK2 included in the mTOR pathway [26]. In two separate *in vitro* kinase inhibition assays, RSK2 was inhibited by SC-1. Furthermore, when the COLO 205 tumor line was exposed to a known RSK2 inhibitor (SL0101 at low concentration), increased clonogenic activity was observed; thereby implying RSK2 might, at least, play a role in promoting clonogenicity. However, the *in vitro* EC_50_ of 2.5 µM exceeds the concentration of SC-1 (0.1 µM) utilized in all studies presented here. This contradiction may be explained by a slow catabolic rate of SC-1 or transport parameters that concentrate SC-1 intracellularly to levels approaching the measured EC_50_. Alternatively, the two values resulted from assays that are of short term (30 min for *in vitro* kinase) or long term (5 day exposure for clonogenicity studies) and a strict comparison is limited. Finally, it also remains possible that the concentration presented to the tumor lines influences several signal transduction pathways in subtle ways to affect a survival outcome and RSK2 is only one such target.

In conclusion, the results presented in this report demonstrate that it is feasible to develop an *in vitro* model of CSC biology with a small molecule in existing tumor lines. The results found in this proof of concept study support a role for the effector function of signal transduction pathways in establishing the CSC phenotype.

## Materials and Methods

### Ethics Statement

All animals were handled in strict accordance with good animal practice as defined by the National Institutes of Health (NIH) Guide for the Care and Use of Laboratory Animals, and the Public Health Service (PHS) Policy on Humane Care and Use of Laboratory Animals. Animal protocols were approved by the NCI-Frederick Animal Care and Use Committee (ACUC). The experiments were conducted under ACUC protocol number 08-082.

### Reagents

Antibodies utilized in the FACS analyses were as follows: FITC conjugated anti-P-glycoprotein (BD Biosciences, 1∶8 dilution), PE conjugated anti-CD133 (Miltenyi Biotec, 1∶4 dilution), PE-Cy7 conjugated anti-CD24, (BD Biosciences, 1∶8 dilution), APC conjugated anti-CD166 (BD Biosciences, 1∶8 dilution), Alexa700 conjugated anti-CD326 (EpCam, BD Biosciences, 1∶8 dilution), Pacific blue conjugated anti-CD44 (BD Biosciences, 1∶8 dilution). Antibodies for immunoblotting and dilutions were as follows: anti-OCT4 (Millipore, 1∶1000), anti-actin (Sigma Aldrich, 1∶4000) and anti-phospho ERK 1/2 and anti-total ERK 1/2 (Cell Signaling Technologies, 1∶1000).

SC-1 was a kind gift of Dr. Peter Schultz, Scripps Research Institute, La Jolla, CA. The compound was stored in 100% DMSO (−20°C) and diluted (at least 10,000 fold) into media prior to any particular assay. SL0101 was purchased from Tocris Biosciences ((Ellisville, MO) and prepared in a similar fashion.

### Tumor Lines and Cell Culture

The seven colon cancer lines (COLO 205, HCC-2998, HCT-15, HCT-116, HT29, KM12, SW-620) utilized in this study were obtained from the NCI60 Anti-cancer Drug Screen (Development Therapeutics Program/DCTD/NCI, Frederick, MD http://dtp.nci.nih.gov) and have been characterized for genetic identity to exclude contamination [36]. All cultures were maintained under standard conditions (RPMI 1640 supplemented with 2 mM glutamine and 5% fetal bovine serum, 5% CO_2_, 37°C).

In all experiments, each tumor line was cultured in 60 mm^2^ tissue culture treated dishes at an initial concentration of 62,500/ml (total 4 ml) before addition of 0.1 µM SC-1 or an equivalent amount of diluent (DMS0) the next day. Five day exposures were conducted. The final concentration utilized for treatment for all tumor lines was determined by evaluating a range of SC-1 concentrations (0.01 to 10 µM) for sphere formation and any cytotoxic effects in the HCT-116 tumor line. Cell viability was routinely evaluated with the trypan blue exclusion test and was always >95% for concentrations at or below 0.1 µM.

### Sphere Formation Assay

The 7 colon tumor lines were seeded in 60 mm^2^ tissue culture treated dishes (Falcon) at low density (62,500 cells/ml) in complete media containing 5% FBS one day prior to addition of 0.1 µM SC-1 (final cell concentration 31,250 cells/ml). Photomicrographs were prepared on day 1 and day 5 following compound treatment utilizing a Zeiss XM inverted microscope and CCD cooled-camera imaging system. Nonadherent spheres were defined as floating, circular structures with well defined borders. Occasionally, exceptionally large spheres re-adhered to the tissue culture surface. Aggregates of floating cells or grape-like clusters were not considered spheres.

### FACS Analysis

A BD FACsAria (BD Biosciences, San Jose, CA) was utilized for FACS analysis according to standard procedures. The instrument was equipped with a 488 nm 100 mW blue laser, a 638 nm 30 mW red laser, and a 405 nm 50 mW violet laser. The software package Diva 5.0 (BD Biosciences) was used for collection and analysis of data. The alignment of the three lasers in the flow cell was verified prior to running tumor cell samples by running Rainbow Beads (Spherotech). The six colors being used in the analysis were then compensated to account for spectral overlap emitted by the fluorochromes within each laser as well as across the lasers using a semi-automated process. This was done using compensation beads (BD Biosciences) that were coated with anti-mouse antibodies. The beads were incubated with each individual antibody used in the six-color panel for 15 min at room temperature. An unstained tube was also prepared. The unstained beads along with beads stained for each fluorochrome were run individually, recording 5000 events. The data was then run through the Diva software compensation matrices and the amount of “spectral bleed” from other fluorochromes into the fluorochrome of interest’s channel was determined and automatically compensated.

Treated and untreated cells (1×10^6^ cells/condition) were harvested with trypsin EDTA (Invitrogen), washed, and then labeled with a six antibody cocktail (antibodies against the following epitopes: CD44, CD24, CD133, CD326, CD166, and P-glycoprotein in PBS without calcium and magnesium supplemented with 2% FBS) or appropriate isotype control antibodies. An unstained control was also prepared. The specific amounts needed for each labeled antibody were determined through titration studies. All cells were incubated with antibody for 15 minutes on ice and in the dark. The cells were then washed, resuspended in buffer, and analyzed on the FACsAria. For each experimental condition, the isotype control tube was evaluated first and 10,000 events were collected. Forward vs. side scatter gates were then established to eliminate likely debris. Further gating was determined for each conjugated isotype control antibody such that 1% of the population was considered a positive staining event. Cells stained with specific labeled antibodies were then evaluated and 10,000 events collected.

### Cell Cycle Analysis

Approximately 5×10^6^ cells were washed with PBS, resuspended in 5 ml of 4°C 70% ethyl alcohol (ETOH) and then incubated at 4°C for 30 min. The sample was washed again before the addition of 0.5 ml PBS containing 0.2 mg/ml of RNAse A. The sample was then incubated at 37°C for 15 minutes. Next, 0.5 ml propidium iodide (0.05 mg/ml in PBS) was added and incubated at 4°C in the dark for 15 min. The sample was then analyzed on the FACScan (Becton Dickinson, San Jose, CA) flow cytometer. Modfit software evaluated DNA content with an automatic module to determine the fraction of cells in the cell cycle compartments.

### Soft Agar Cloning Assay

The soft agar colony formation assay has been previously described [37]. In brief, for each treatment condition, triplicate 35 mm^2^ dishes were coated with a 1 ml base layer containing 0.7% agarose (Seaplaque: FMC Corp., Rockland, ME). On day 0, cells were dissociated, washed once in growth medium, and subcultured by layering various cell concentrations in 0.5 ml culture medium containing 0.3% agarose onto the base coat. Culture dishes were transferred to a refrigerator (4°C) for 15 min, to room temperature for 10 min, and then to culture incubators. An upper layer of 0.5 ml culture medium was applied on day 0. Cultures were stained with MTT (1 mg/ml) for visualization and imaging at multiple time intervals (day 7 and 14).

Cumulative counts of colonies (diameters, >60 µm) and cumulative volumes of CFU (diameters >120 µm) were determined with an Omnicon FAS-II (Bausch & Lomb, Rochester, NY) following calibration with an Omnicon test plate 3 and polystyrene microspheres embedded in soft agar matrix. The evaluable region of each culture dish (35 contiguous fields equivalent to 51% of the cell layer culture volume) was analyzed. Selective scoring of viable cell groups was achieved by adjustment of the instrument detection threshold to exclude images of nonstained cellular material and debris. The data was analyzed with Lotus Symphony software.

### *In vivo* Subcutaneous Xenograft Model

Six week old female NOD.SCID mice were randomly assigned to each of 20 treatment arms (n = 5/group) [38]. Treated and control colon tumor lines were enzymatically harvested and counted prior to dilution in RPMI 1640 (at 10, 100, 1,000, 10,000 cells/inoculum) for subcutaneous injection into the axillary region using a volume of 1 ml/mouse. Positive control groups were established such that tumor formation occurred in all mice in approximately one week thus confirming that all tumor lines studied were tumorigenic. Cell inocula for the positive control groups ranged from 1×10^6^ to 2.5×10^6^. Cell viability of the cell stocks was assessed by trypan blue exclusion prior to dilution for injection and was routinely found to be >95%. Tumor formation was monitored weekly using caliper measurements and the tumor mass was calculated as weight in mg  = 1/2×(tumor length)×(tumor width)^2^. All experiments were terminated at 120 days or when tumor weight exceeded 2000 mg for any individual mouse. In selected studies, tumor cells were resuspended in Matrigel (50% concentration, BD Biosciences) prior to injection.

### Protein Electrophoresis and Immunoblotting

Standard protein electrophoretic techniques were followed using previously published protocols [39]. For these studies, the colon lines were cultured for 5 days with and without treatments, harvested with trypsin (0.25% trypsin 0.5 mM EDTA, Invitrogen), counted and lysed with a whole cell lysis buffer (10 mM Tris, 1.0 mM EDTA, 50 mM NaCl, 0.5% deoxycholate, 0.5% NP-40, 0.5% SDS, 1.0 mM PMSF, and aprotonin and a cocktail of phosphatase and protease inhibitors). The whole cell lysate was heated to 100°C for 10 min and then fractionated (1000 g, 10 min, 4°C). Protein concentrations were measured with the Pierce BCA Protein Assay. Twenty to 40 µg of protein lysate per lane were loaded onto 10% SDS polyacrylamide gels (Ready-Gels, Biorad) with Laemmli buffer. For immunoblotting, the primary antibodies were diluted in Tween/Tris wash buffer (0.1 mM Tris-HCl pH 7.5, 2.5% Tween-20, 150 mM NaCl) and 5% BSA or nonfat dry milk and incubated overnight at 4°C. Visualization of specific bands was accomplished with an alkaline phosphatase-labeled secondary anti-immunoglobulin antibody and the ECF Chemifluorescent reagent (GE Amersham). Pixel intensity of the imaged bands was measured using STORM (GE Amersham) and Image Quant Software. Normalization for loading was accomplished by reprobing with an anti-actin antibody, quantification of pixel intensity and data normalization.

### *In vitro* Kinase Inhibition Assay

The RSK2 *in vitro* kinase inhibition assay utilized was a modification of protocols established by Nguyen et al. [40]. Additional protocols that confirmed the results depicted in Figure 4 and evaluated kinase inhibitory activity in a random selection of protein kinases (Table 3) were conducted at Reaction Biology Corporation (Malvern, PA). This was a miniaturized ^33^P-based screening assay [41].

### Statistical Analyses

Chi square tests and nonparametric conditional estimate for the Cochran Mantel-Haenzel common odds ratio were performed for the limiting dilution tumorigenicity studies. In the later case, the tumor lines were used as the stratification variable and the test evaluated whether the tumor take rate was equal in the two groups (control and treated). The purpose of combining data across distinct colon tumor lines was to aggregate small changes that if summed, may represent a heterogeneous population of patients with colon tumors, similar to analyses in clinical trials.

Student’s t tests were performed on the results from the clonogenicity studies. Student’s paired t tests (two-tailed) were performed on the time to first tumor appearance in the tumorigenicity studies and changes in expression of surface antigens.

## Supporting Information

Figure S1 **Chemical Structure of SC-1.**
(PDF)

Figure S2 **Effect of SC-1 on Tumor Formation in Colon Tumor Lines.** Tumor cell lines were treated with 0.1 µM SC-1 for 5 days prior to injection into NOD SCID mice at cell inocula indicated (10, 100, 1000, and 10000 cells/injection). No protein support was provided. The fraction without tumors in each group was determined at 120 days following inoculation. Negative results for tumor lines showing no effect under these conditions are provided in Table 1 in the main text. Control: black squares, Treated: white squares.
(PPTX)

Figure S3 **Frequency Estimates of Tumor Initiating Ability for Cumulative Tumor Take Rate and Selected Colon Tumor Lines.** A. Percent of mice without tumors (Fraction Negative) was plotted against the variable cell inoculum size (10, 100, 1,000, 10,000 cells per injection) for control and SC-1 treated colon tumor lines (n = 7). Frequency estimates were calculated from Taswell [42] and was increased approximately 2-fold for the SC-1 treated population. B. Frequency estimates and confidence intervals were plotted for each treatment group for the cumulative data derived from limiting dilution tumorigenicity assay. No statistically significant results were found. C. Percent of mice without tumors (Fraction Negative) was plotted against the variable cell inoculum size (10, 100, 1000, 10000 cells per injection) for the control and most sensitive SC-1 treated COLO 205, HCT-116, and HT29 colon tumor lines. Frequency estimates were calculated and were the highest for the SC-1 treated population. D. Frequency estimates and confidence intervals were plotted for each treatment group for the combined results of COLO 205, HCT-116, and HT29 treated tumor lines derived from limiting dilution tumorigenicity assay. There was a statistically significant difference for the control and SC-1 treated comparison (p = 0.008).
(TIF)

Figure S4 **Effect of SC-1 on Distribution of Colon Tumor Lines across the Cell Cycle.** HCT-116 tumor line was incubated with the treatments under study and harvested on day 5 prior to analysis of the cell cycle compartments as described in the Materials and Methods. Black bars: control treated; Gray bars: SC-1 treated. None of the experimental treatments altered the distribution of the cells across the cell cycle (n = 2).
(PDF)

Figure S5 **SC-1 Increased Sphere Formation in HT29 Tumor Line Grown in Serum Free Media and Low Attachment Vessels.** HT29 tumor line was cultured at 0.5–8 cells/µl in serum free media (RPMI 1640 containing EGF (20 ng/ml), bFGF (10 ng/ml) and B27 supplement) one day prior to addition of SC-1 (0.1 µM). The number of spheres per well was counted on Day 1 (A) and Day 5 (B) following treatment. Statistically significant effects (*p<0.05) for SC-1 treatment were found at all conditions where spheres formed. A representative experiment of 3 is shown here.
(PDF)

Table S1 **SC-1 Decreased Cell Growth for 7 Colon Tumor Cell Lines.** After a five day exposure to 0.1 µM SC-1, the seven colon tumor lines were evaluated for changes in cell number and viability. There was a statistically significant decrease in cell number but >95% viability.
(DOC)
