# Supplementary figures and images for: A Small Molecule (Pluripotin) as a Tool for Studying Cancer Stem Cell Biology: Proof of Concept
Source: PLoS One. 2013 Feb 21;8(2):e57099. doi: 10.1371/journal.pone.0057099 (PMC3578829; doi:10.1371/journal.pone.0057099)

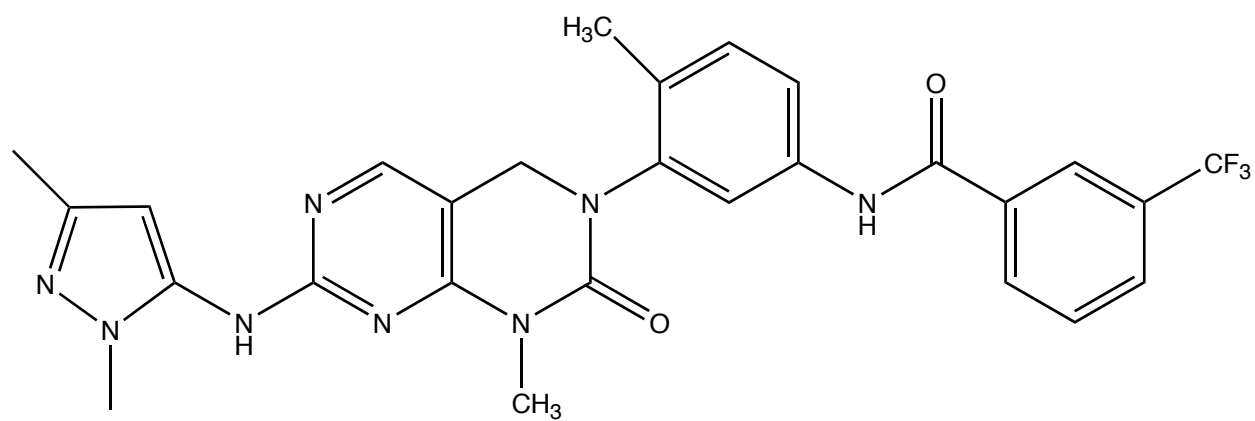

Supplement: Figure S1 — Chemical Structure of SC-1. (PDF) [file pone.0057099.s001.pdf]

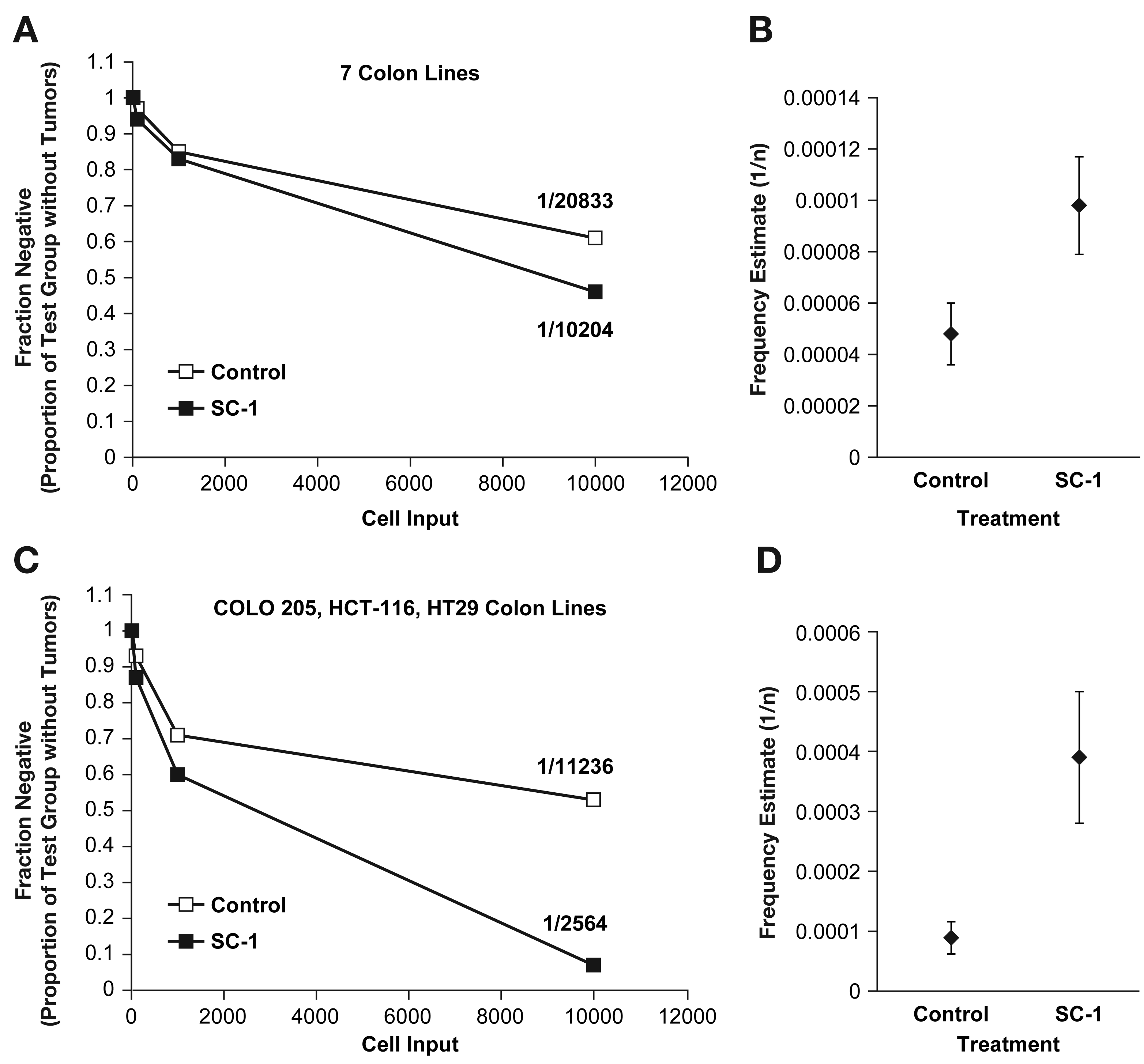

Supplement: Figure S3 — Frequency Estimates of Tumor Initiating Ability for Cumulative Tumor Take Rate and Selected Colon Tumor Lines. A. Percent of mice without tumors (Fraction Negative) was plotted against the variable cell inoculum size (10, 100, 1,000, 10,000 cells per injection) for control and SC-1 treated colon tumor lines (n = 7). Frequency estimates were calculated from Taswell [42] and was increased approximately 2-fold for the SC-1 treated population. B. Frequency estimates and confidence intervals were plotted for each treatment group for the cumulative data derived from limiting dilution tumorigenicity assay. No statistically significant results were found. C. Percent of mice without tumors (Fraction Negative) was plotted against the variable cell inoculum size (10, 100, 1000, 10000 cells per injection) for the control and most sensitive SC-1 treated COLO 205, HCT-116, and HT29 colon tumor lines. Frequency estimates were calculated and were the highest for the SC-1 treated population. D. Frequency estimates and confidence intervals were plotted for each treatment group for the combined results of COLO 205, HCT-116, and HT29 treated tumor lines derived from limiting dilution tumorigenicity assay. There was a statistically significant difference for the control and SC-1 treated comparison (p = 0.008). (TIF) [file pone.0057099.s003.tif]

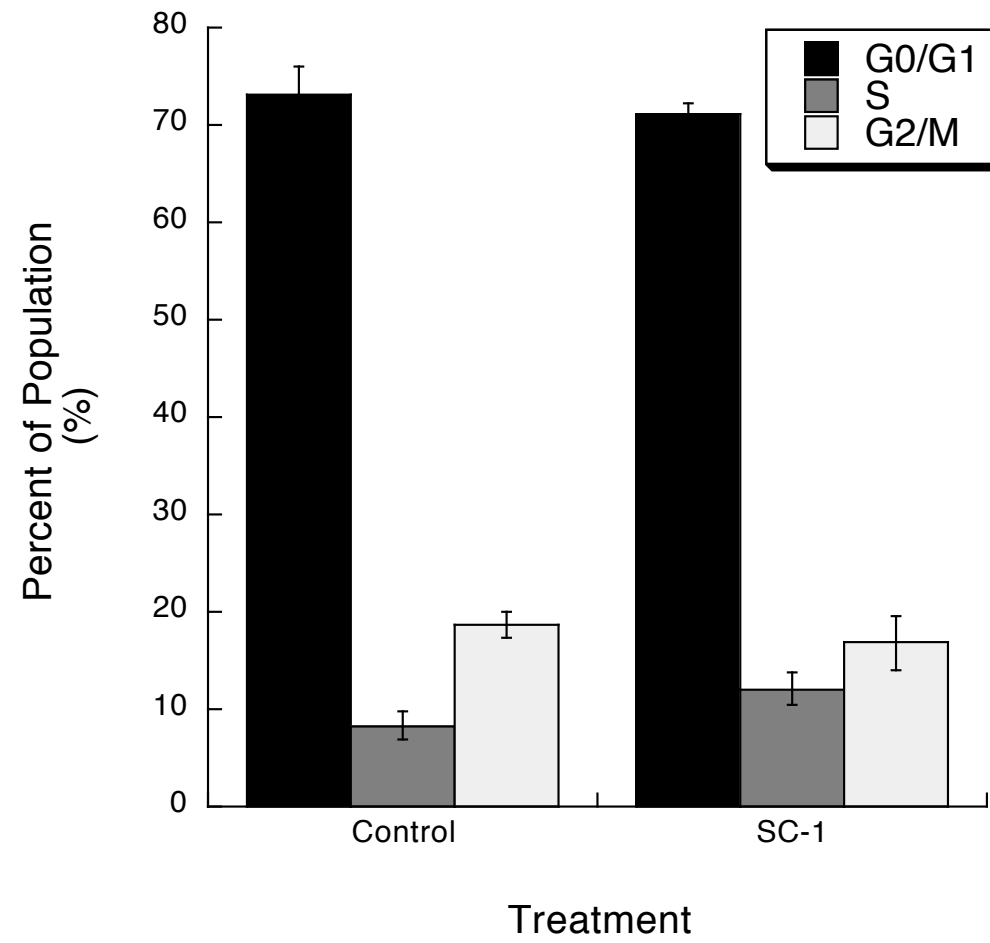

Supplement: Figure S4 — Effect of SC-1 on Distribution of Colon Tumor Lines across the Cell Cycle. HCT-116 tumor line was incubated with the treatments under study and harvested on day 5 prior to analysis of the cell cycle compartments as described in the Materials and Methods. Black bars: control treated; Gray bars: SC-1 treated. None of the experimental treatments altered the distribution of the cells across the cell cycle (n = 2). (PDF) [file pone.0057099.s004.pdf]

A

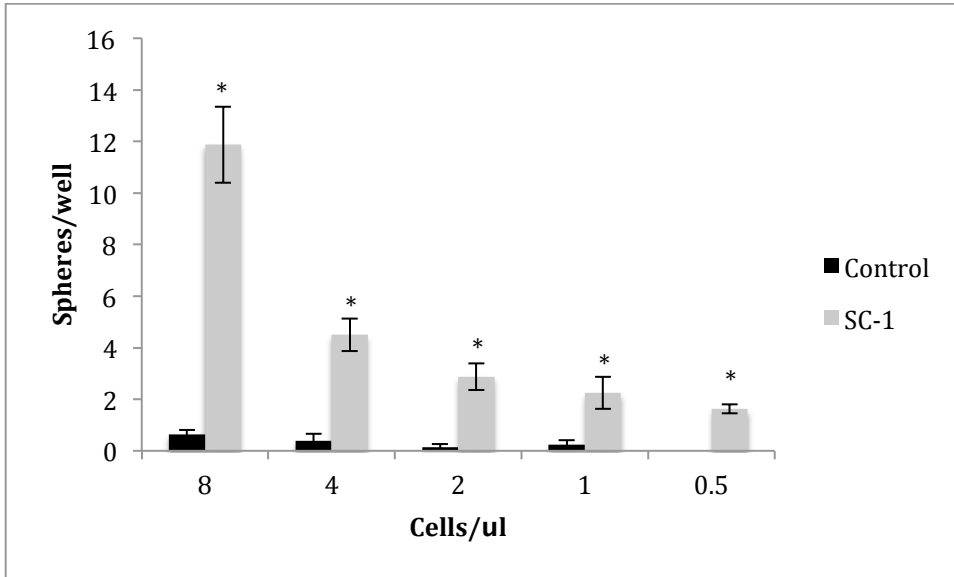

B

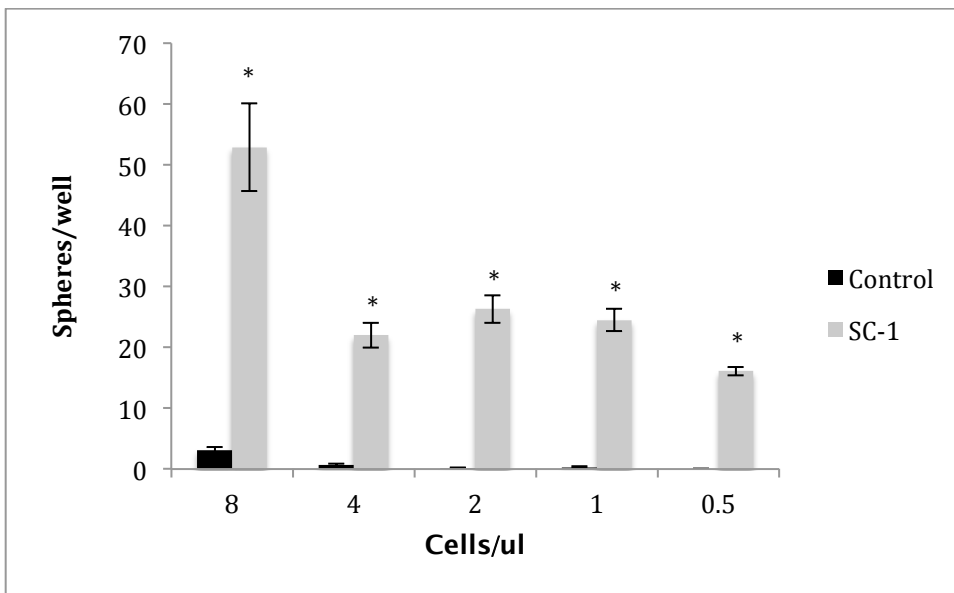

Supplement: Figure S5 — SC-1 Increased Sphere Formation in HT29 Tumor Line Grown in Serum Free Media and Low Attachment Vessels. HT29 tumor line was cultured at 0.5–8 cells/µl in serum free media (RPMI 1640 containing EGF (20 ng/ml), bFGF (10 ng/ml) and B27 supplement) one day prior to addition of SC-1 (0.1 µM). The number of spheres per well was counted on Day 1 (A) and Day 5 (B) following treatment. Statistically significant effects (*p<0.05) for SC-1 treatment were found at all conditions where spheres formed. A representative experiment of 3 is shown here. (PDF) [file pone.0057099.s005.pdf]
