# Supplementary material for: A Small Molecule (Pluripotin) as a Tool for Studying Cancer Stem Cell Biology: Proof of Concept
Source: PLoS One. 2013 Feb 21;8(2):e57099. doi: 10.1371/journal.pone.0057099 (PMC3578829; doi:10.1371/journal.pone.0057099)
Supplement: Table S1 — SC-1 Decreased Cell Growth for 7 Colon Tumor Cell Lines. After a five day exposure to 0.1 µM SC-1, the seven colon tumor lines were evaluated for changes in cell number and viability. There was a statistically significant decrease in cell number but >95% viability. (DOC) [file pone.0057099.s006.doc]

Table S1. SC-1 Decreased Cell Growth for 7 Colon Tumor Cell Lines*

|  | **Treatment** | Control |  | SC-1@ |  |
| --- | --- | --- | --- | --- | --- |
| Cell Line |  |  | Fold Change from Initial Inocula |  |  |
|  |  |  |  |  |  |
| COLO 205 |  | 11.2 |  | 2 |  |
|  |  |  |  |  |  |
| HCC-2998 |  | 3.4 |  | 4 |  |
|  |  |  |  |  |  |
| HCT-15 |  | 28.4 |  | 19.8 |  |
|  |  |  |  |  |  |
| HCT-116 |  | 62 |  | 31 |  |
|  |  |  |  |  |  |
| HT29 |  | 30.6 |  | 17.5 |  |
|  |  |  |  |  |  |
| KM12 |  | 38.2 |  | 20.6 |  |
|  |  |  |  |  |  |
| SW-620 |  | 56.3 |  | 27.5 |  |

*Each colon cell line was seeded on day 0 with 0.25 x 10^6^ cell/60 mm^2^ Petri dish. Compound (0.1 μM) was added on day 1 and cells were enzymatically harvested 5 days later. Viability was routinely examined by trypan blue exclusion and found to be >95% in all cases.

@Significant decrease between treated and control groups, p=0.02 by paired Student’s t test (n=7).
